# LncRNA MEG3 influences the proliferation and apoptosis of psoriasis epidermal cells by targeting miR-21/caspase-8

**DOI:** 10.1186/s12860-019-0229-9

**Published:** 2019-10-28

**Authors:** Hai-Yan Jia, Kai Zhang, Wen-Jing Lu, Gui-Wen Xu, Jian-Fen Zhang, Zhan-Li Tang

**Affiliations:** 1 Department of Dermatology, Qilu Hospital of Shandong University, No.107, West Wenhua Road, Jinan, 250012 Shandong Province People’s Republic of China; 2 Department of Neurosurgery, Shengli Oilfield Central Hospital, Dongying, 257000 People’s Republic of China

**Keywords:** LncRNA MEG3, Psoriasis, miR-21, Caspase-8

## Abstract

**Background:**

It was reported that microRNA-21(miR-21) was differentially expressed in the keratinocytes of psoriasis patients, and it may influence the apoptosis and proliferation of cells. The role of lncRNA maternally expressed gene3 (MEG3), a competing endogenous RNAs of miR-21, in the progression of psoriasis remains unclear. We aimed to unfold the influence of MEG3 and miR-21 on the proliferation and apoptosis of psoriasis epidermal cells.

**Methods:**

50μg/L TNF-α was used to treat HaCaTs and NHEKs cells for 24 h, and then different experiments were conducted. qRT-PCR were applied for measuring the mRNA level of MEG3, miR-2, and caspase-8, and the protein expression of caspase-8 was measured with western blotting. Flow cytometry was used for assessing apoptosis. Cell proliferation was detected using MTT and colony formation assays. Dual luciferase reporter assay was applied for confirming the binding site between MEG3 and miR-21, miR-21 and Caspase-8.

**Results:**

A cell model for in vitro studying the role of MEG3 in psoriasis pathophysiology was established using HaCaT and HHEKs. MEG3 was significantly down-regulated in HaCaT, HHEKs, and psoriatic skin samples. MEG3 inhibits proliferation and promotes apoptosis of Activated-HaCaT (Act-HaCaT) and Activated-HHEKs (Act- HHEK) by regulating miR-21, and the binding site between MEG3 and miR-21 was identified. We also found that miR-21 could inhibit the level of caspase-8 and identified the binding site between caspase-8 and miR-21. Some down-stream proteins of caspase-8, Cleaved caspase-8, cytc, and apaf-1 were regulated by miR-21 and MEG3.

**Conclusion:**

MEG3/miR-21 axis may regulate the expression of caspase-8, and further influence the proliferation and apoptosis of psoriasis keratinocyte, Act-HaCaT and Act- HHEK. Therefore, our findings may provide a new thought for the study of pathogenesis and treatment of psoriasis.

## Background

Psoriasis is a chronic, relapsing, and papulosquamous dermatitis affecting 3% of population in the world [1]. Psoriasis is characterized by abnormal hyperproliferation of the epidermis, and the pathogenesis of it may be caused by genetic, immune, and inflammatory factors. In the level of histology, psoriasis is characterized by apoptosis delay and increased proliferation of keratinocytes [2]. Therefore, the study about regulating the proliferation and apoptosis of keratinocytes may provide promising therapeutic target for psoriasis.

MicroRNAs are endogenous, small, non-coding RNAs that regulate gene expression through binding to the 3′untranslated region of target mRNAs [3]. MicroRNA-21(miR-21) is considered as an oncogene, and the up-regulation of it has been reported in a wide range of solid and leukaemic cancers such as colon, gastric, breast and pancreatic cancers [4]. Overexpression of miR-21 also inhibited apoptosis in osteosarcoma. Significant high level of miR-21 was observed in psoriasis lesion compared with non-lesional skin from psoriasis patients [3]. However, influence of miR-21 on the over proliferation of keratinocytes have not been reported.

Long non coding RNAs (lncRNAs) belongs to non-protein-coding RNA, and it plays a key role in many human diseases by regulating miRs. Maternally expressed gene3 (MEG3), which encodes a lncRNA, is an imprinted gene belonging to the DLK1-MEG3 locus located on chromosome 14q32.3 in humans [5]. Significant lower expression of MEG3 was confirmed in several types of cancers, such as gastric cancer, nonsmall cell lung cancer and gallbladder cancer. Overexpression of MEG3 could suppress proliferation and promote apoptosis in some type of tumor cells [6–8]. At present, there are only few studies about the role of lncRNA in the pathogenesis of psoriasis. Furthermore, the function of lncRNA MEG3 in psoriatic keratinocytes is not yet clear.

Some reports indicated that MEG3 affected proliferation, apoptosis, and metastasis of gastric and cervical cancer cells by regulating miR-21 [5, 9]. It was reported that physically MEG3 was linked with miR-21, and miR-21 was downregulated following ischemia, which was opposite to MEG3 [10]. However, the influence of MEG3 on miR-21 during the proliferation and apoptosis of psoriasis epidermal cells remains unclear.

In this study, we identified the differential expression of lncRNA MEG3 in the overactivated keratinocytes. Additionally, we found the influence of lncRNA MEG3 on the proliferation and apoptosis of overactivated keratinocytes. Then the relationship between lncRNA MEG3 and the expression of miR-21 in psoriasis was investigated. We found that lncRNA MEG3 affected apoptosis and proliferation of HaCaT and HHEKs by regulating the expression of miR-21 and caspase-8.

## Result

### The differential expression of lncRNA MEG3 and the excessive proliferation of keratinocytes

**Fig. 1:**
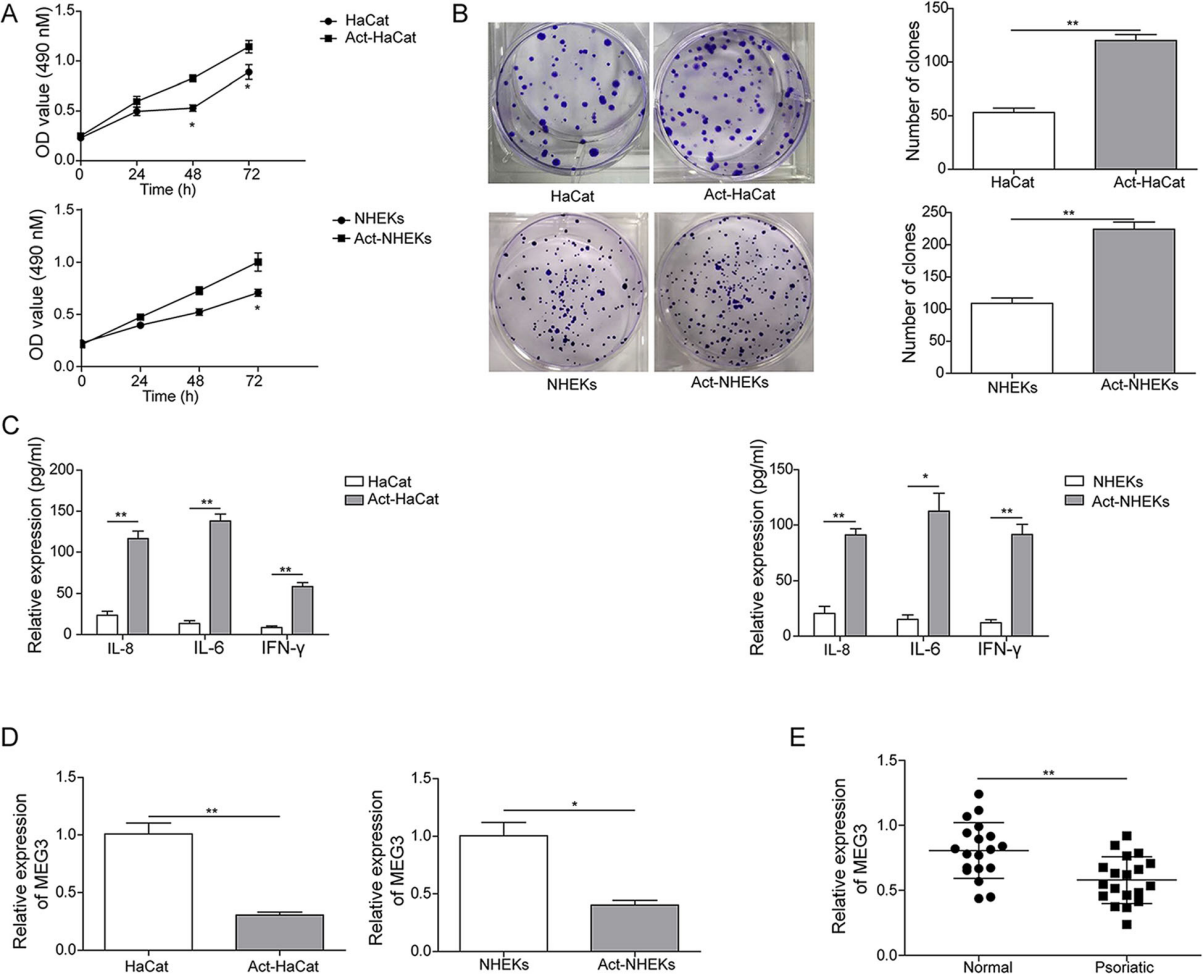
The differential expression of lncRNA MEG3 in the excessive proliferation of keratinocytes. **a** Cell proliferation was measured by MTT assay; **b** Cell viability was measured by clone formation assay; **c** Expression of IL-6, IL-8, and INF-γ in Act-HaCaT and Act-HHEK was measured by Elisa; **d** mRNA expression of MEG3 in cells was measured by qRT-PCR. **e** The expression of MEG3 in normal skin samples and psoriatic skin samples was detected by qRT-PCR. All the results were shown as mean ± SD (*n* = 3), and the experiments were repeated three times

Firstly, we investigated the proliferation of HaCaT, Act-HaCaT, HHEKs, and Act-HHEK cells by MTT and clone formation assays after TNF-α treatment. The proliferation rate of Act-HaCaT and Act-HHEK cell was significantly higher than HaCaT and HHEKs, respectively at different time points measured by MTT assay, which was in line with the characteristic of keratinocytes in psoriasis (Fig. 1a). Clone formation assay (Fig. 1b) was applied to confirm the different proliferation rate between Act-HaCaT and HaCaT, Act-HHEK and HHEKs, and the result of which was identical to MTT assay. It was reported that the serum levels of IL-8 and IL-6 were significantly higher in psoriasis patients compared to healthy subjects [11]. Therefore, we measured the concentrations of IL-8, IL-6, INF-γ, and found that their contents in Act-HaCaT and Act-HHEK was remarkably higher than HaCaT and HHEKs (Fig. 1c), which is in line with previous findings. In the present study, the MEG3 expression in Act-HaCaT and Act-HHEK was statistically lower than HaCaT and HHEKs measured by qPCR (Fig. 1d). We have detected the expression of LncMEG3 in normal skin samples and psoriatic skin samples by RT-q PCR. The results indicated that compared with normal skin samples, the expression of LncMEG3 in psoriatic skin samples was significantly down-regulated. (Fig. 1e). Therefore, the model we constructed has the characteristics of psoriatic keratinocytes, and the different expression of lncRNA MEG3 was found, which was in line with clinical results [12].

### LncRNA MEG3 suppresses proliferation and promotes apoptosis of act-HaCaT and act-HHEK

**Fig. 2:**
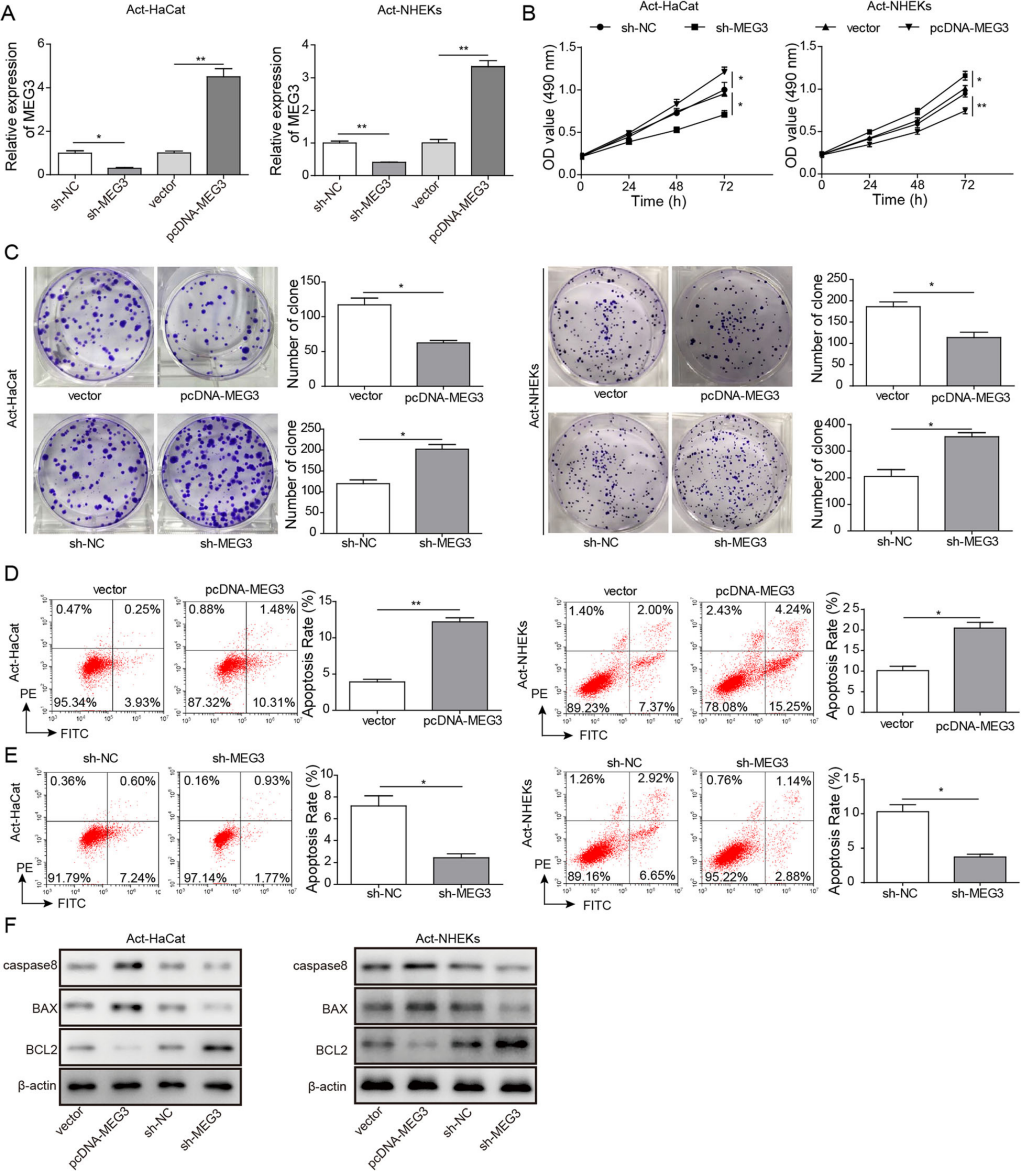
LncRNA MEG3 inhibited proliferation and promotes apoptosis of Act-HaCaT and Act-HHEK. **a** Transfection efficiency of sh-MEG3 and pcDNA-MEG3 in cells was measured by qRT-PCR; **b** Influence of MEG3 expression on the proliferation of Act-HaCaT and Act-HHEK was measured by MTT assay; **c** Influence of MEG3 expression on the proliferation of Act-HaCaT and Act-HHEK was measured by clone formation assay; **d** Overexpression of MEG3 promoted the apoptosis of Act-HaCaT and Act-HHEK; **e** Knockdown of MEG3 inhibited the apoptosis of Act-HaCaT and Act-HHEK. **f** caspase-8, Bax, and Bcl-2 protein levels after transfecting vector, pcDNA-MEG3, sh-NC, and sh-MEG4, respectively. All the results were shown as mean ± SD (*n* = 3), and the experiments were repeated three times

To unfold the influence of lncRNA MEG3 on proliferation and apoptosis of Act-HaCaT and Act-HHEK, sh-MEG3, sh-NC, vector, and pcDNA-MEG3 were transfected respectively. We detected the transfection efficiency of MEG3 by qRT-PCR. The results showed that compared with sh-NC, the expression of MEG3 in Act-HaCaT and Act-NKEK cells was decreased significantly in sh-MEG3 group. Compared with vector group, the expression of MEG3 in pcDNA-MEG3 group was increased significantly, indicating that sh-MEG3 and pcDNA-MEG3 were successfully transfected into Act-HaCaT and Act-NKEK cells (Fig. 2a). We found that the proliferation of Act-HaCaT and Act-HHEK increased significantly after treatment by sh-MEG3 transfection compared with the other three groups. Therefore, downregulated lncRNA MEG3 could remarkably promote cell proliferation, and overexpressing MEG3 could markedly inhibit cell proliferation. Additionally, transfection with pcDNA-MEG3 significantly inhibit the proliferation of Act-HaCaT and Act-HHEK (Fig. 2b). Similar results were observed by the method of clone formation assay (Fig. 2c). Therefore, MEG3 may inhibit the proliferation of psoriatic keratinocytes with excessive proliferation.

We further investigated the effect of lncRNA MEG3 on apoptosis of Act-HaCaT and Act-HHEK. The results of flow cytometry analysis indicated that transfection with pcDNA-MEG3 significantly increased the ratio of apoptosis to ~ 10% compared with group vector (Fig. 2d). While, transfection with sh-MEG3 remarkably decreased the ratio of apoptosis to ~ 1% compared with group sh-NC (Fig. 2e). Meanwhile, we detected the apoptosis related proteins, caspase-8, Bcl-2, and Bax, through western blotting. Our study indicated that transfection with pcDNA-MEG3 significantly increased expression of caspase-8 and Bax, which promote apoptosis of Act-HaCaT and Act-HHEK, and decreased the level of Bcl-2, which inhibits apoptosis (Fig. 2f). While, transfection with sh-MEG3 caused opposite effect. Therefore, the influence of MEG3 on apoptosis of Act-HaCaT and Act-HHEK corresponded with its influence on cell proliferation.

### LncRNA MEG3 inhibits the expression of miR-21

**Fig. 3:**
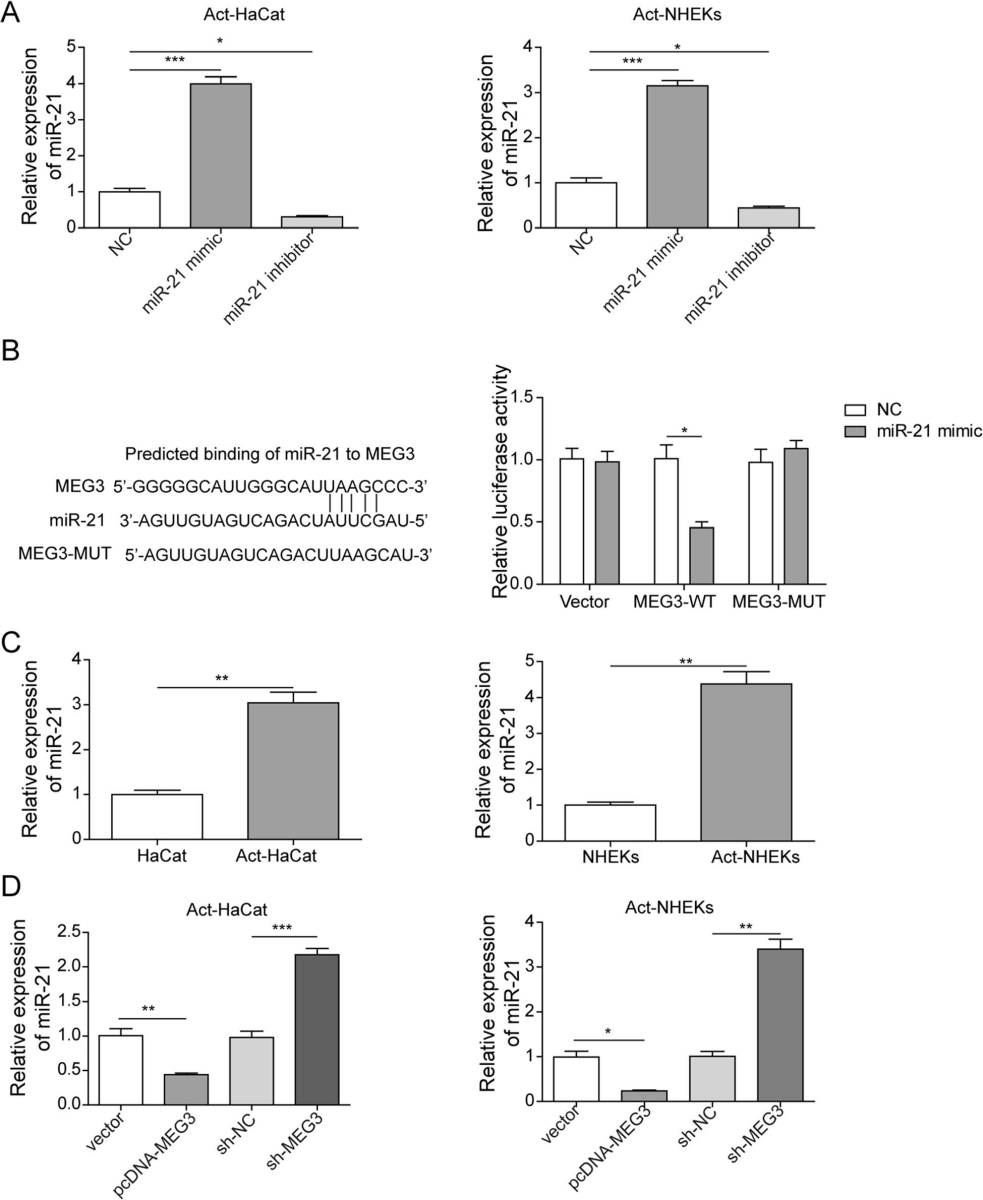
LncRNA MEG3 suppressed the expression of miR-21 in Act-HaCaT and Act-HHEK. **a** Transfection efficiency of miR-21 mimic and inhibitor in Act-HaCaT and Act-HHEK was measured by qRT-PCR; **b** Prediction of the binding site between miR-21 and MEG3; **c** Transfection with miR-21 mimic and MEG3 WT presented dual luciferase inhibition; **d** The mRNA level of miR-21 was detected after transfection with sh-NC, sh-MEG3, vector, and pcDNA-MEG3, respectively. All the results were shown as mean ± SD (*n* = 3), and the experiments were repeated three times

Previous studies reported that differential expression of miR-21 in psoriasis was observed [3]. Therefore, we further investigated whether there are direct interactions between them. qRT-PCR was used to detect the transfection efficiency of miR-21. The results showed that in Act-HaCaT and Act-NKEK cells, compared with NC group, the expression level of miR-21 in the miR-21 mimic group was significantly increased, while decreased in the miR-21 inhibitor group, indicating that the miR-21 mimic and inhibitor were transfected successfully (Fig. 3a). The results of dual luciferase reporter assay indicated that only transfection with miR-21 mimic and MEG3 WT presented dual luciferase inhibition, Meanwhile, transfection of mutant MEG3 and miR-21 mimic did not cause the decrease of fluorescence, which suggested that there is a direct binding site between miR-21 and MEG3 (Fig. 3b and Fig. 3c).

To unfold how lncRNA MEG3 regulate miR-21 expression, we firstly validate differential expression of miR-21 in Act-HaCaT, HaCaT, Act-NKEK, and NKEK using qPCR. The levels of miR-21 in Act-HaCaT and Act-NKEK were remarkably higher than HaCaT and NKEK, respectively. Meanwhile, transfection with pcDNA-MEG3 could remarkably decrease the level of miR-21, and transfection with sh-MEG3 brought opposite effect (Fig. 3d). Therefore, there is a negative regulatory relationship between lncRNA MEG3 and miR-21, and lncRNA MEG3 should be a competitive endogenous substance of miR-21 in psoriasis.

### LncRNA MEG3 affects the cell proliferation and apoptosis by regulating miR-21

**Fig. 4:**
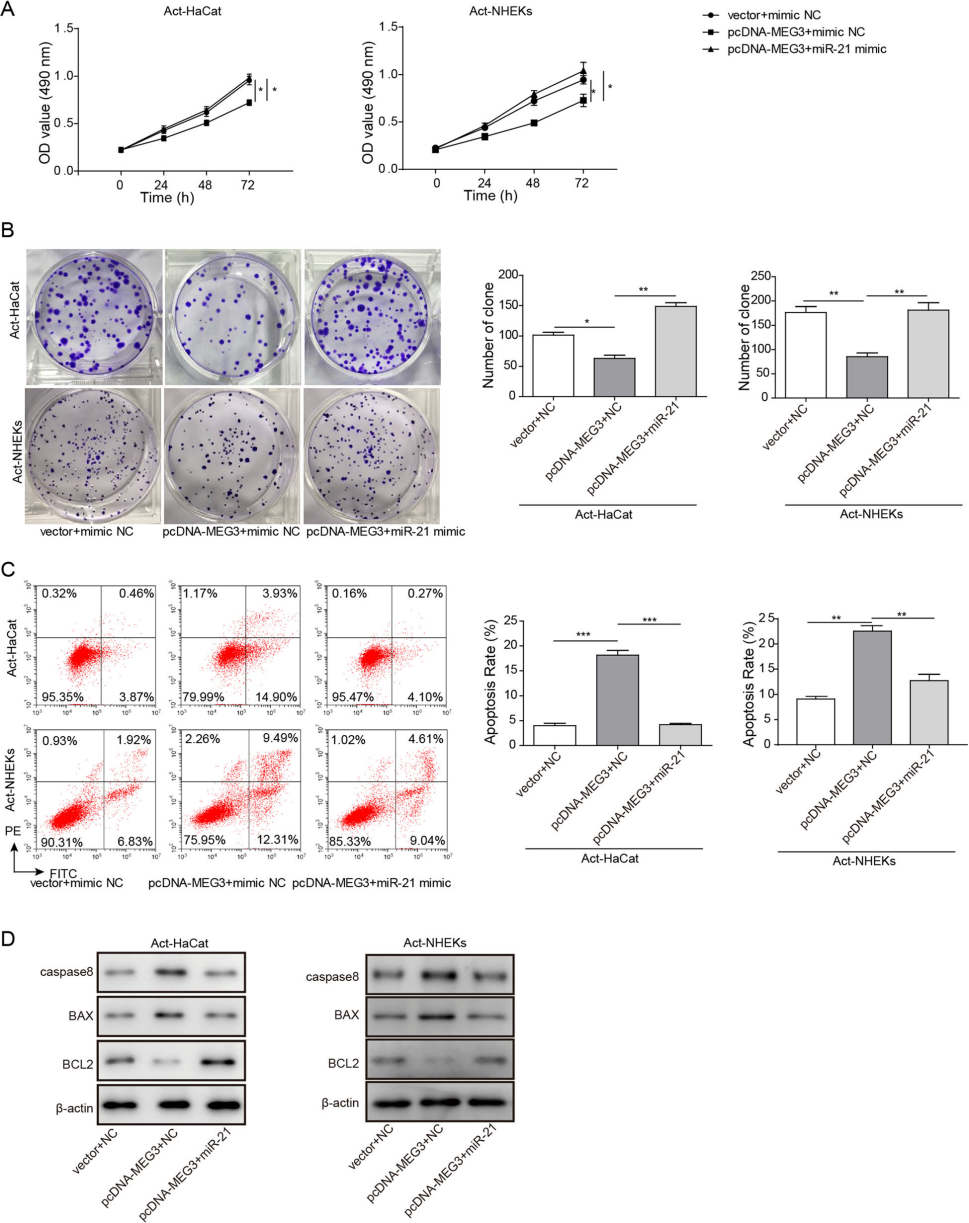
LncRNA MEG3 affected the proliferation and apoptosis of Act-HaCaT and Act-HHEK by regulating miR-21. **a** Cell proliferation of Act-HaCaT and Act-HHEK was measured by MTT assay after transfection with vector+miR- NC, pcDNA-MEG3 + miR- NC, and pcDNA-MEG3 + miR-21 minmic, respectively; **b** Cell proliferation of Act-HaCaT and Act-HHEK was measured by clone formation assay after transfection with vector+miR- NC, pcDNA-MEG3 + miR- NC, and pcDNA-MEG3 + miR-21 minmic, respectively; **c** Influence of co-transfection using pcDNA-MEG3 + miR- NC and miR-21inhibitor on apoptosis of Act-HaCaT and Act-HHEK; **d** Influence of transfection with vector+miR-NC, pcDNA-MEG3 + miR- NC and pcDNA-MEG3+ miR-21 mimic on the expression of caspase-8, Bax, and Bcl-2, respectively. All the results were shown as mean ± SD (*n* = 3), and the experiments were repeated three times

In order to explore the regulatory relationship between lncRNA MEG3 and mRNA-21 in cell proliferation and apoptosis, we detected the proliferation and apoptosis of cells after different treatments. We found that the cell transfected by vector+NC or pcDNA-MEG3 + miR-21 mimic proliferated significantly faster than groug pdDNA-MEG3 + mimic+NC measured by MTT. Presumably, MEG3 may affect proliferation of Act-HaCaT and Act-NKEK through miR-21, and the adjustment is negative (Fig. 4a). Meanwhile, similar conclusions were observed in the clone formation assay after 10 days’ culture (Fig. 4b). Flow cytometery analysis was applied to unfold the role of MEG3 and miR-21 in the cell apoptosis. Transfection with pcDNA-MEG3 significantly increased apoptosis compared with transfection with vector+mimic+NC. However, transfection with pcDNA-MEG3 + miR21-mimic remarkably decreased apoptosis (Fig. 4c). The results of flow cytometery analysis indicated that the increase of apoptosis ratio caused by pcDNA-MEG3 could be reversed by miR-21 mimic. We also found that transfection with pcDNA-MEG3 + NC significantly increased expression of caspase-8 and Bax, which promote apoptosis and decreased the level of Bcl-2, which inhibits apoptosis. While, transfection with pcDNA-MEG3 + miR-21 mimic caused opposite effect (Fig. 4d). Therefore, the regulatory relationship between lncRNA MEG3 and miR-21 affected apoptosis and cell proliferation. Overexpression of MEG3 can inhibit cell proliferation, and miR-21 can reverse the effect of MEG3 on cell proliferation.

### miR-21 inhibits the expression of caspase-8

**Fig. 5:**
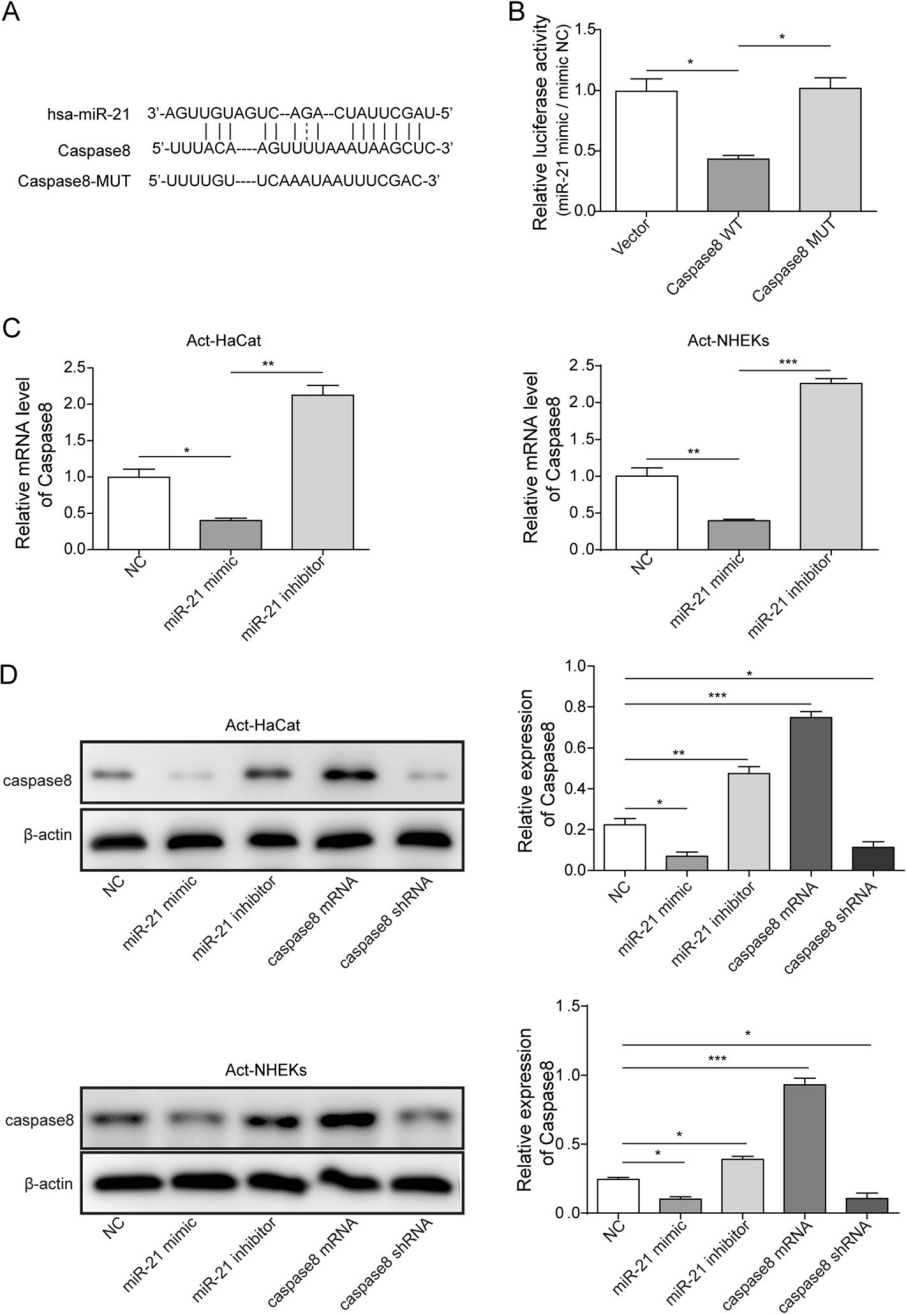
miR-21 inhibited the expression of caspase-8 in Act-HaCaT and Act-HHEK. **a** Prediction of the binding site between miR-21 and caspase-8; **b** Transfection with miR-21 mimic and caspase-8 WT decreased the relative luciferase activity (miR-21/miR-NC); **c** Transfection with miR-21 mimic significantly decreased the expression of caspase-8; **d** The expression of caspase-8 measured by western blotting after transfection with miR-21 inhibitor, miR-21 mimic, caspase-8 mRNA, and caspase-8 shRNA, respectively. All the results were shown as mean ± SD (*n* = 3), and the experiments were repeated three times

To ensure the influence mechanism of miR-21 on apoptosis and cell proliferation, we analyzed the binding sites between caspase-8 and miR-21 by bioinformatics. The binding site between miR-21 and caspase-8 was predicted firstly (Fig. 5a). The result of dual luciferase reporter assay indicated that the relative luciferase activity (miR-21/miR-NC) decreased only on the condition that miR-21 and caspase-8 were transfected simultaneously (Fig. 5b). This finding suggested that direct binding site exist between miR-21 and caspase-8. The expression of caspase-8 mRNA decreased significantly after transfecting miR-21 mimic. However, significant increase of caspase-8 was observed after treatment by miR-21 inhibitor (Fig. 5c). After transfecting miR-21 mimic, miR-21 inhibitor, caspase-8 mRNA, and caspase-8 shRNA in Act-HaCaT and Act-NKEK respectively, the expression of caspase-8 in protein level was measured by western blotting. The protein expression of caspase-8 decreased markedly after transfecting miR-21 mimic, but transfection with miR-21 inhibitor reversed this trend, which was in line with qPCR assay (Fig. 5d). Transfection with caspase-8 mRNA significantly up-regulated caspase-8 expression, but caspase-8 shRNA brought opposite effect (Fig. 5d). Therefore, there are direct binding sites between miR-21 and caspase-8 mRNA, which could directly affect the expression of caspase-8.

### LncRNA MEG3 affects the proliferation and apoptosis through regulating miR-21 expression and further influencing caspase-8

**Fig. 6:**
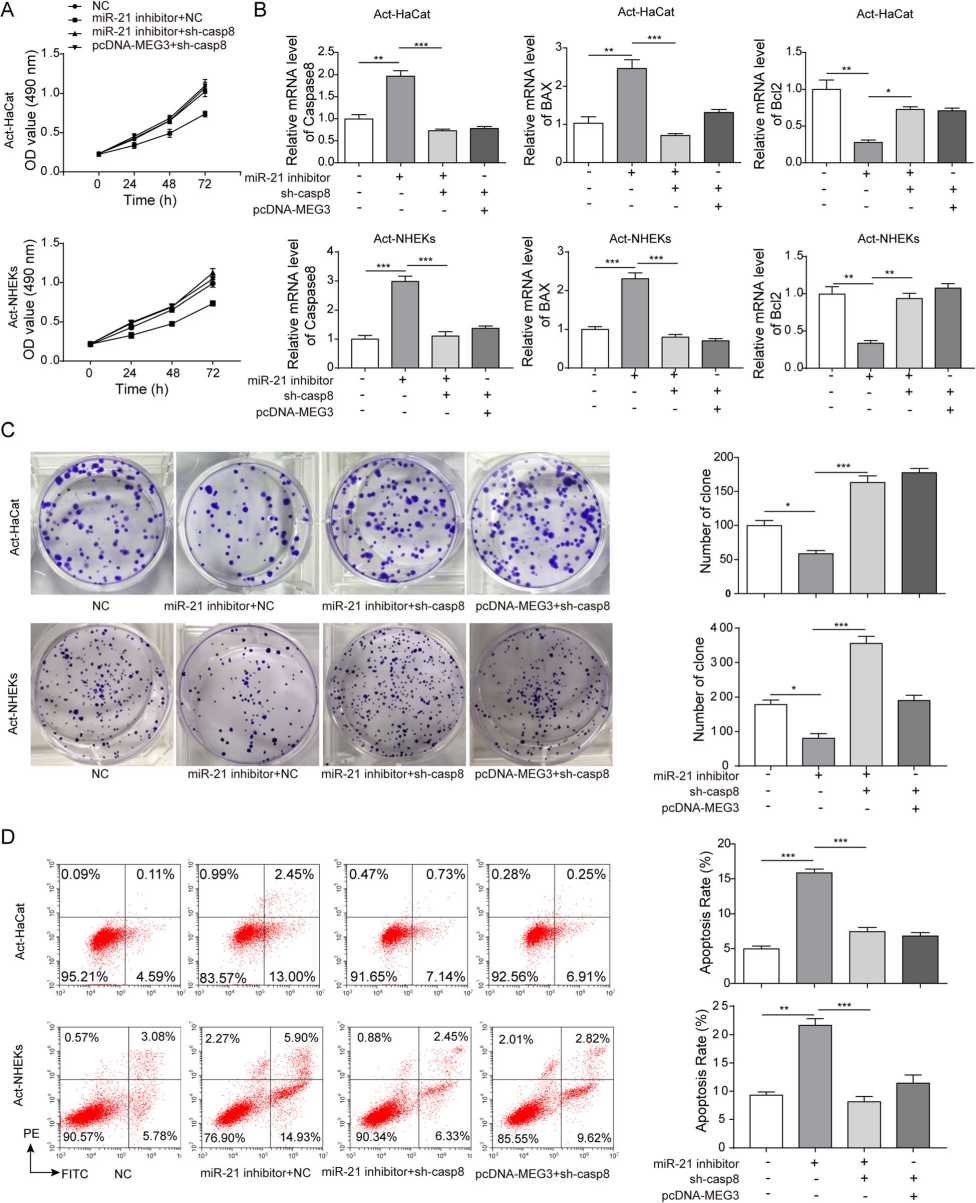
LncRNA MEG3 affected the proliferation and apoptosis of Act-HaCaT and Act-HHEK by regulating miR-21 expression and further influencing caspase-8. **a** The proliferation of Act-HaCaT and Act-HHEK was measured by MTT assay after transfection with miR-21 inhibitor+sh-NC, miR-21 inhibitor+sh-caspase-8, and pcDNA-MEG3 + sh-caspase-8, respectively. **b** The proliferation of Act-HaCaT and Act-HHEK was measured by colony formation assay after transfection with miR-21 inhibitor+sh-NC, miR-21 inhibitor+sh-caspase-8, and pcDNA-MEG3 + sh-caspase-8, respectively. **c** The apoptosis ratio of Act-HaCaT and Act-HHEK was measured after transfection with miR-21 inhibitor+sh-NC, miR-21 inhibitor+sh-caspase-8, and pcDNA-MEG3 + sh-caspase-8, respectively. **d** The mRNA expression of caspase-8, Bax, and Bcl-2 in Act-HaCaT and Act-HHEK after transfection with miR-21 inhibitor+sh-NC, miR-21 inhibitor+sh-caspase-8, and pcDNA-MEG3 + sh-caspase-8, respectively. All the results were shown as mean ± SD (*n* = 3), and the experiments were repeated three times

To illustrate the effect of lncRNA MEG3/miR-21/caspase-8 on proliferation of psoriatic keratinocytes we transfected NC, miR-21 inhibitor+NC, miR-21 inhibitor+sh-caspase-8, pcDNA-MEG3 + sh-caspase-8 respectively in Act-HaCaT and Act-NKEK. The results of MTT and clone formation assays indicated that transfection with miR-21 inhibitor+NC significantly inhibited cell proliferation, but transfection with miR-21 inhibitor+sh-caspase-8 could restore the ability of cell proliferation (Fig. 6a and Fig. 6c). Transfection with miR-21 inhibitor+NC remarkably increased apoptosis of Act-HaCaT and Act-NKEK compared with group NC, but transfection with miR-21 inhibitor+ caspase-8 shRNA or pcDNA-MEG3+ caspase-8 shRNA effectively reversed this result (Fig. 6b). This finding suggested that miR-21 could regulate apoptosis through caspase-8, and overexpression of MEG3 could regulate the level of miR-21.

**Fig. 7:**
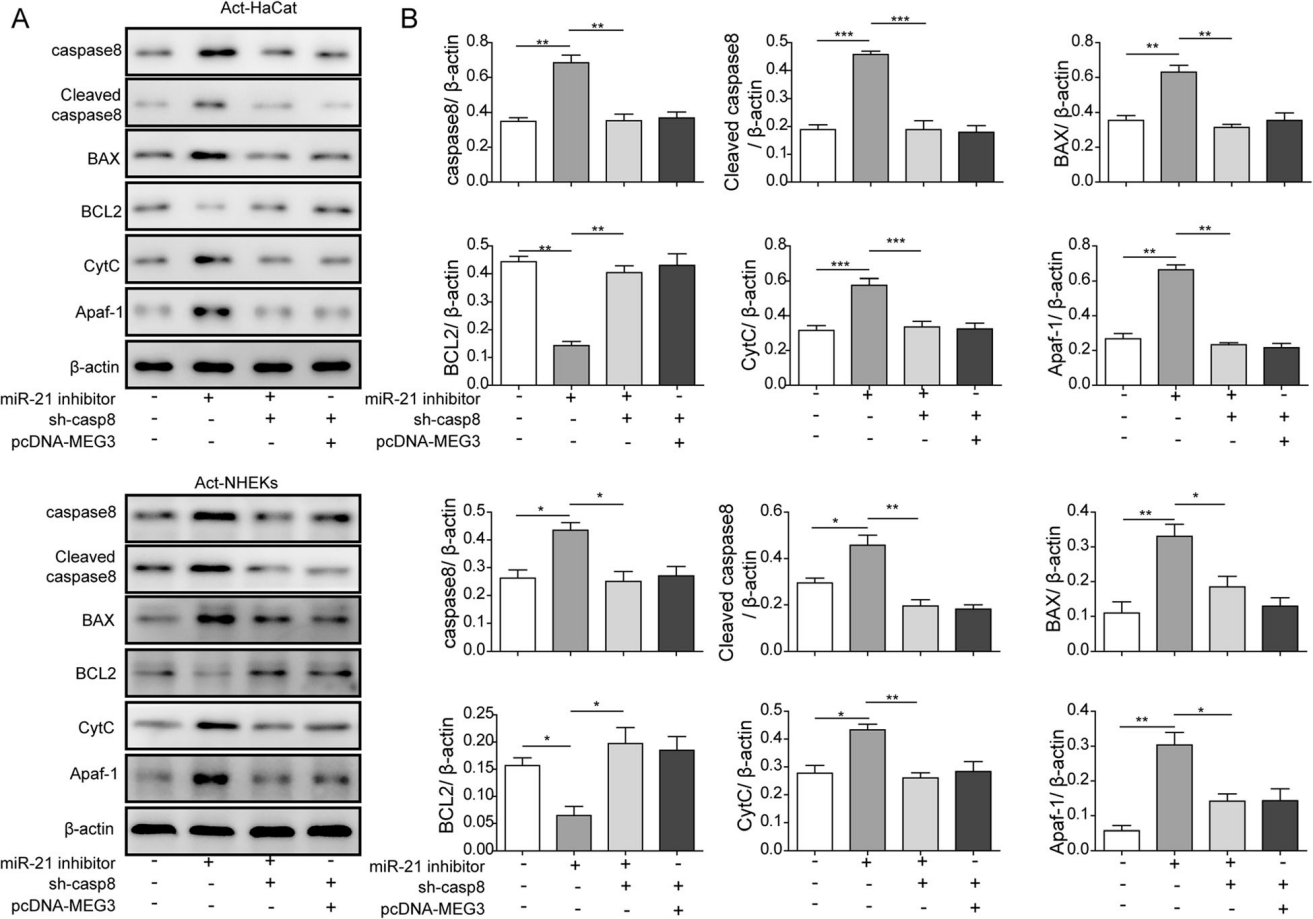
The apoptosis proteins and Cyt-C in Act-HaCaT and Act-HHEK cells were measured by Westernblot. **a** The protein expression of caspase-8, Cleaved caspase-8, CvtC, Apaf-1, Bax, and Bcl-2 in Act-HaCaT and Act-HHEK after transfection with miR-21 inhibitor+sh-NC, miR-21 inhibitor+sh-caspase-8, and pcDNA-MEG3 + sh-caspase-8, respectively. **b** Protein gray value analysis of caspase-8, Cleaved caspase-8, CvtC, Apaf-1, Bax, and Bcl-2 in Act-HaCaT and Act-HHEK after transfection with miR-21 inhibitor+sh-NC, miR-21 inhibitor+sh-caspase-8, and pcDNA-MEG3 + sh-caspase-8, respectively. All the results were shown as mean ± SD (*n* = 3), and the experiments were repeated three times

Transfection with miR-21 inhibitor+NC remarkably increased mRNA and protein expression of caspase-8 and Bax, which promote apoptosis of Act-HaCaT and Act-NKEK, and decreased the level of Bcl-2, which inhibits apoptosis (Fig. 6d, Fig. 7 a and Fig. 7b). However, transfection with miR-21 inhibitor+ caspase-8 shRNA or pcDNA-MEG3+ caspase-8 shRNA significantly reversed these trends. Transfection with miR-21 inhibitor+NC significantly increased caspase-8, Bax, Cyt-C, Apaf-1, and decreased Bcl-2, but miR-21 inhibitor+ caspase-8 shRNA and pcDNA-MEG3+ caspase-8 shRNA could reverse these changes above (Fig. 7a and Fig. 7b). These findings suggest that overexpression of lncRNA MEG3 could inhibit the proliferation of psoriatic keratinocytes and promote apoptosis by inhibiting the level of miR-21 and promoting the expression of caspase-8.

## Discussion

Psoriasis is a common inflammatory skin disorder, affecting around 3% of the population in the world. It is characterized by over-proliferation and/or disturbed differentiation of keratinocytes [12]. In this study, we found the differential expressions of MEG3 and miR-21 in over-proliferation keratinocytes, and demonstrated the regulation between MEG3 and miR-21. Meanwhile, we confirmed that miR-21 influence the proliferation and apoptosis of cell by modulating caspase-8, and this finding may provide a new insight for the treatment of psoriasis.

It was reported that MEG3 could serve as a cancer suppressor and is able to induce the apoptosis of gliomas cells and leukemia cells [13, 14] . Meanwhile, MEG3 may play a vital role in other activities, such as repairing processes of bone marrow derived mesenchymal stem cells [15] and angiogenesis after ischemic stroke [16]. It was reported that differential expression of MEG3 was observed in psoriasis [12]. However, studies about influence of MEG3 on psoriatic keratinocyte are not common. In this study, we proved that MEG3 could inhibit the proliferation of psoriatic keratinocyte and promote cell apoptosis.

LncRNA functions as an endogenous competitive RNA of microRNAs. Some reports unfolded the regulation between lncRNA and microRNA in cancer therapy thoughIt was reported that MEG3 may enhance cisplatin sensitivity in non-small cell lung cancer through modulating miR-21 [17, 18]. MEG3 regulated Imatinib resistance in chronic myeloid leukemia by inhibiting miR-21 [19]. It was also found that LncRNA CASC7 could affected apoptosis and cell proliferation of colon cancer cells by targeting miR-21 [20] . The finding in this study that MEG3 influenced the cell proliferation and apoptosis by regulating miR-21 could be a potential therapeutic target for the treatment of psoriasis.

Caspase activation mechanism is an important signaling pathway causing apoptosis [21, 22]. The caspases are composed of effectors (caspase 3, 6, and 7) and initiators (caspase 8 and 9) [23, 24]. The cleaved caspase-8 subsequently promotes apoptosis via proteolytic activation of these downstream effectors [25, 26]. It was reported that elevated Cyt-C and Apaf1 could trigger intrinsic apoptosis via executive molecular caspase-9 and caspase-3 [27]. Meanwhile, it was reported that some lncRNAs promoted the apoptosis of tumor cells through targeting caspase 8 [28, 29]. In this study, miR-21 inhibitor remarkably increased the apoptotic cell rate, whereas caspase-8 silencing partly reversed the decreased apoptosis. Therefore, miR-21 might inhibit apoptosis via targeting caspase-8. Taking into consideration the regulation of miR-21 by MEG3, we concluded that MEG3/miR-21 axis may modulate cell proliferation and apoptosis by targeting caspase-8.

## Conclusion

In this study, we studied the influence of MEG3 on psoriasis keratinocytes in vitro, which may provide a new thought for the study of pathogenesis and treatment of psoriasis. However, the insufficiency of this study is no investigation of in vivo model research, which is because of that psoriasis is a multi-factor induced disease. The study of lncRNA in psoriasis animal model is our ongoing work.

## Methods

### Patients and healthy controls

Nineteen cases (9 males and 10 females) and 19 controls (10 males and 9 females) were recruited from Qilu Hospital of Shandong University (July 2018 to January 2019) (Table 1). Patients did not receive systemic drugs, phototherapy or externally applied drugs during the last 3 months prior to sample collection. The patients without any autoimmune disorders or systemic disorders were diagnosed as having psoriasis by at least two dermatologists. Healthy controls in this study were individuals without psoriasis, any family history of psoriasis (including first- to third-degree relatives) and other autoimmune or systemic diseases. Same questionnaire, including gender, age, the skin lesion type, family history, age at onset and other epidemiological data, was used to collect clinical and demographic information from both cases and controls. This study was approved by the Ethics Committee of Shandong University, Qilu Hospital (Jinan, China). All patients enrolled in the present study provided written informed consent.

**Table 1:** The count of clinical characteristics in psoriasis

Subphenotypes	Case (*N* = 19)	Control (*N* = 19)
Gender		
Male	9	10
Female	10	9
Age of onset		NA
> 40 years	8	
≤ 40 years	11	
Types of disease		NA
Acute guttate	6	
Chronic plaque	13	
Family history		NA
Positive	5	
Negative	14	

*NA* not available; *s.d*. standard deviation

### Cell culture

HaCaT and NHEKs cell lines (American Type Culture Collection, USA) were chosen in this study. Cells were cultured in Eagle’s Minimum Essential medium (EMEM; Gibco, USA) supplemented with 10% newborn calf serum (NCS) and streptomycin and penicillin (All from Biochrom KG, Berlin, Germany) before treatment with TNF-α (Peprotech, USA) at 37 °C in humidified air of 5% CO_2_. For experiments, HaCaTs and NHEKs cells (5 × 10^4^ cells/ml) in good logarithmic growth state were seeded in a culture dish, and cultured in the incubator. After incubation with 50 μg/L TNF-α (10 ng/ml) for 24 h, protein or RNA was extracted from Act-HaCaT and Act- HHEK cells. For apoptosis and proliferation assays, NCS concentration was 1%.

### Cell transfection

GenePharma Co., Ltd. (Shanghai, China) designed and synthesized sh-MEG3, pcDNA-MEG3, vector+mimic NC, pcDNAMEG3 + mimic NC, pcDNA-MEG3 + miR-21 mimic, miR-21 inhibitor, miR-21 mimic, caspase-8 mRNA, and caspase-8 shRNA. After stimulation with TNF-α (10 ng/mL) for 24 h, cells were plated on 60-mm dishes and cultured for 24 h. Cell transfection and cotransfection were conducted with Lipofectamine 2000 (Invitrogen) according to instruction.

### Cell viability assay

The proliferation of cells was measured by MTT assay. Cells were seeded into 96-well plates, and cultured 1–3 days. After incubation with TNF-α (10 ng/mL) for 24 h, MTT reagent (Sigma, St. Louis, MO, USA) was added to the cells. After 4 h incubation the supernatant was removed and 200 μL DMSO was added. The optical density of each well at 450 nm was detected after 2 h incubation by Multiskan EX (Thermo, Finland). Each assay was performed in triplicate.

### Flow cytometery analysis

Flow cytometry was used to measure cell apoptosis by Annexin V-fuoresecin isothiocyanate (FITC) apoptosis measurement kit (BD Biosciences, United State). Cells were stimulated with TNF-α (10 ng/mL) for 24 h firstly, and then collected and washed two times by cold PBS. 10^6^ cells were suspended in 200 μL binding buffer containing 5 μL propidium iodide (PI) and 10 μL Annexin V-FITC. Then incubate cells in the dark for 30 min, and the cells were detected through flow cytometry. Apoptotic rate was scored by quantifying early apoptosis (Annexin V-FITC+ PI-) and late apoptosis or necrosis cells (Annexin V-FITC+ PI+). Flow cytometry data were plotted and analyzed by the fluorescence activated cell-sorting (FACS-Vantage) system using Cell Ouest software (Becton-Dickinson, San Jose, CA, USA) within 1 h after staining.

### Clone formation assay

Cells (2 × 10^2^ per well) were seeded in 6-well plates and were cultured in complete media for 2 weeks. After incubation with TNF-α (10 ng/mL) for 24 h, media was removed, cells were washed twice in PBS and stained by crystal violet (Sigma-Aldrich, MO, USA) for 60 min at room temperature. Colonies were counted by inverse microscope (Nikon, Tokyo, Japan). Colonies of > 50 μm in size were counted by Image J. Final results were shown as an average of three independent assays.

### RNA isolation and real-time PCR

Total RNA was isolated from cells and tissues with TRIzol reagent (Invitrogen Life Technologies, USA). After the isolation of RNA, the ratio between A260/A230 and A260/A280 was measured by nanodrop 2000 Microultraviolet spectrophotometer (1011 U, Nanodrop, USA), and the quality and quantity of RNA were measured. 500 ng RNA was reverse-transcribed into cDNA with the Primer Script RT reagent kit (Takara Bio, China). Real-time PCR was performed with SYBR Premix Ex TaqTM II kit (Takara Bio, China). The primers used for MEG3, miR-21, Caspase-8, Bax, and Bcl-2 were listed as follows: (1) MEG3: forward: 5′-CTGCCCATCTACACCTCACG-3′ and reverse: 5′-CTCTCCGCCGTCTGCGCTAGGGGCT-3′; (2) miR-21: forward: 5′-CAGATCAGCCGCTGTCACA-3′ and reverse: 5′-TGCCCACCGCACAC-3′; (3) Caspase-8: forward: 5′-ACCGAGATCCTGTGAATGGA-3′ and reverse: 5′-TGCTTTCCCTTGTTCCTCCT-3′; (4) Bax: 5′- CCACCAGCTCTGAACAGATC-3′ and reverse: 5′-CAGCTTCTTGGTGGACGCAT-3′; (5) Bcl-2: 5′-TGGGATGCCTTTGTGGAACT-3′ and reverse: 5′-GAGACAGCCAGGAGAAATCA-3′; (6) GAPDH: forward: 5′-CATACAAGGTCATCTCCAACGC-3′ and reverse: 5′-AAGGTCCGTCAACAGTCTTCTG-3′; (7) U6: forward: 5′-GTGACCTTTATTGCG ACATCCACT-3′ and reverse: 5′-CTTCTGAAACAC GAGTCATATGTG GT-3′. GAPDH and U6 were used as the the internal reference of MEG3 and miR-21, respectively. Each sample was replicated three times with no RT and no template control included. Data were analyzed by comparing cycle threshold values. The relative expression of target genes was calculated using the 2^-∆∆Ct^ method. ∆∆Ct = ∆Ct _experiment_ - ∆Ct _control_, ∆Ct = Ct _target gene_ - Ct _control gene_. The fold change between the experimental group and the control group = 2^-∆∆Ct^. Ct is the number of amplified cycles when the real-time fluorescence intensity of the reaction reaches a set threshold.

### Protein extraction and western blot analysis

Primary antibodies used in this study were purchased from Abcam (Hong Kong, China). Total proteins of cells were prepared using lysis buffer. Equal amounts of protein were loaded on an SDS-PAGE and then transferred electrophoretically to PVDF membrane (Millipore, USA). After blocking with TBST (5% milk), the membranes were incubated overnight with primary antibody (1:1000) at 4 °C. After washing and incubation, the membranes were incubated with secondary antibody (1:2000) in TBST. Immunodetection was conducted through ECL Plus detection system (Millipore, USA). The density of the bands was measured using Image J software.

### Dual luciferase reporter assay

Cells (2.0 × 10^4^) were plated in a 24-well plate. Cells were cotransfected with pmirGLO-MEG3-WT or pmirGLO- MEG3-MUT and miR-21 mimics with lipofectamine 2000 (Invitrogen life technologies, USA). Luciferase activity was detected 48 h after transfection with dual-luciferase reporter gene assay kit (Promega, USA) according to manufacturer instruction.

### Statistical analysis

Data were presented as the mean ± SE, and compared using Student’s t-test. *P*-value < 0.05 was considered as statistically significant difference.

## Data Availability

All data generated or analysed during this study are included in this published article [and its supplementary information files].

## Abbreviations

Act-HaCaT: Activated-HaCaT
Act-NKEK: Activated-NKEK
lncRNAs: Long non coding RNAs
MEG3: Maternally expressed gene3
miR-21: MicroRNA-21
NCS: Newborn calf serum
